# Supplementary material for: Assessment of Respiratory Health Symptoms and Asthma in Children near a Drying Saline Lake
Source: Int J Environ Res Public Health. 2019 Oct 11;16(20):3828. doi: 10.3390/ijerph16203828 (PMC6843482; doi:10.3390/ijerph16203828)
Supplement: Supplementary file 1 [file ijerph-16-03828-s001.pdf]

International Journal of
Environmental Research and Public Health

MDPI

Supplemental Material

Page

Supplemental Table S1. Prevalence of respiratory symptoms, health care utilization and medication use among school-age participants (N = 357). 2-3

Supplemental Table S2. Impact of wheezing among asthmatic and non-asthmatic participants reporting any lifetime wheeze (N = 126). 4

Supplemental Table S3. Housing characteristics among study participants (N = 357). 5

Supplemental Table S1. Prevalence of respiratory symptoms, health care utilization and medication use among school-age participants (n = 357).

Respiratory Symptoms	N (%)
**Lifetime Wheezing**	
Yes	126 (35.3)
No	213 (59.7)
Missing	18 (5.0)
**Allergies**	
Yes	129 (36.1)
No	203 (56.9)
Missing	25 (7.0)
**Dry Cough**	
Yes	119 (33.3)
No	220 (61.6)
Missing	18 (5.0)
**Dry Cough for More than 3 Weeks**	
Yes	46 (12.9)
No	73 (20.5)
Missing	238 (66.7)
**Dry Cough in the Morning**	
Yes	31 (8.7)
No	313 (87.7)
Missing	13 (3.7)
**Dry Cough at Other Times of the Day**	
Yes	27 (7.6)
No	318 (89.1)
Missing	12 (3.4)
**Congestion**	
Yes	61 (17.1)
No	279 (78.2)
Missing	17 (4.8)
**Bronchitic Symptoms**[a]	
Yes	102 (28.6)
No	255 (71.4)
**Doctor Visit Due to Wheezing**	
Yes	103 (28.9)
No	254 (71.2)
**ER Visit Due to Wheezing**	
Yes	57 (16.0)
No	300 (84.0)
**Medication Use**	
Yes	83 (23.3)
No	267 (74.8)
Missing	7 (2.0)
**Rescue Medication Use**	
Yes	90 (25.2)
No	267 (74.8)
**Control Medication Use**	
Yes	35 (90.2)
No	322 (9.8)
**Nebulizer Use**	
Yes	73 (20.5)
No	284 (79.6)

[a] Bronchitic Symptoms defined as having at least one of the following symptoms 1) daily cough for 3 months in a row, 2) congestion or phlegm for at least 3 months in a row, and 3) bronchitis in the past 12 months.

Supplemental Table S2. Impact of wheezing among asthmatic and non-asthmatic participants reporting any lifetime wheeze (N = 126).

	**Total N = 126 (%)**	**Asthmatics reporting any lifetime wheeze N = 71 (%)**	**Non-asthmatics reporting any lifetime wheeze N = 55 (%)**	**p-value**
**Wheeze in the Past 12 Months**				
Yes	78 (61.9)	53 (74.7)	25 (45.5)	**0.002**
No	44 (34.9)	17 (23.9)	27 (49.1)	
Missing	4 (3.2)	1 (1.4)	3 (5.5)	
**Sleep Disturbance Due to Wheeze in the Past 12 Months**				
Never awakened with wheezing	20 (15.9)	10 (14.1)	10 (18.2)	0.09
Less than one night per week	32 (25.4)	22 (31.0)	10 (18.2)	
One or more nights per week	22 (17.5)	18 (25.4)	4 (7.3)	
Missing	52 (41.3)	21 (29.6)	31 (56.4)	
**Speech Disturbance Due to Wheeze in the Past 12 Months**				
Yes	10 (7.9)	9 (12.7)	1 (1.8)	0.13
No	66 (52.4)	44 (62.0)	22 (40.0)	
Missing	50 (39.7)	18 (25.4)	32 (58.2)	
**Wheeze After Exercise in the Past 12 Months**				
Yes	47 (37.3)	32 (45.1)	15 (27.3)	0.98
No	31 (24.6)	21 (29.6)	10 (18.2)	
Missing	48 (38.1)	18 (25.4)	30 (54.6)	

Supplemental Table S3. Housing characteristics among study participants (N = 357).

	Total N= 357(%)	Asthmatics N = 80 (%)	Non-Asthmatics N = 277 (%)	p-value[a]
**Housing type**				0.91
House	198 (55.5)	46 (57.5)	152 (54.9)	
Apartment	113 (31.7)	25 (31.3)	88 (31.8)	
Mobile home or trailer	35 (9.8)	7 (8.8)	28 (10.1)	
Missing	11 (3.1)	2 (2.5)	9 (3.3)	
**Lived in the same house for whole life**				0.30
Yes	93 (26.1)	25 (31.3)	68 (24.6)	
No	255 (71.4)	55 (68.8)	200 (72.2)	
Missing	9 (2.5)	0 (0.0)	9 (3.3)	
**Gas cooking stove in home**				0.09
Yes	296 (82.9)	62 (77.5)	234 (84.5)	
No	54 (15.1)	17 (21.3)	37 (13.4)	
Missing	7 (2.0)	1 (1.3)	6 (2.2)	
**Length of gas use (N = 296)**				**0.03**
Less than 30 minutes	138 (46.6)	24 (38.7)	114 (48.7)	
Less than 1 hour	111 (37.5)	26 (41.9)	85 (36.3)	
More than 1 hour	28 (9.5)	11 (17.7)	17 (7.3)	
Missing	19 (6.4)	1 (1.6)	18 (7.7)	
**Air conditioner in home**				0.91
Yes	344 (96.4)	78 (97.5)	266 (96.0)	
No	4 (1.1)	1 (1.3)	3 (1.1)	
Missing	9 (2.5)	1 (1.3)	8 (2.9)	
**Water damage in home**				**0.04**
Yes	29 (8.1)	11 (13.8)	18 (6.5)	
No	296 (82.9)	62 (77.5)	234 (84.5)	
Missing	32 (9.0)	7 (8.8)	25 (9.0)	
**Mold**				0.31
Yes	50 (14.0)	14 (17.5)	36 (13.0)	
No	265 (74.2)	57 (71.2)	208 (75.1)	
Missing	42 (11.8)	9 (11.3)	33 (11.9)	
**Musty odor in home**				0.05
Yes	14 (3.9)	6 (7.5)	8 (2.9)	
No	314 (88.0)	66 (82.5)	248 (89.5)	
Missing	29 (8.1)	8 (10.0)	21 (7.6)	
**Carpet in home**				0.27
Yes	213 (59.6)	53 (66.3)	160 (57.8)	
No	132 (37.0)	26 (32.5)	106 (38.3)	
Missing	12 (3.4)	1 (1.3)	11 (4.0)	
**Pet at home**				**0.05**
Furry pet	105 (29.4)	15 (18.8)	90 (32.5)	
Another pet	27 (7.6)	8 (10.0)	19 (6.9)	
None	214 (59.9)	55 (68.8)	159 (57.4)	
Missing	11 (3.1)	2 (2.5)	9 (3.3)	
**Regular contact with farm animal**				0.72
Yes	44 (12.3)	11 (13.8)	33 (11.9)	
No	244 (68.4)	55 (68.8)	189 (68.2)	
Missing	69 (19.3)	14 (17.5)	55 (19.9)	
**Problem with pests at home**				0.70
Insects	83 (23.3)	22 (27.5)	61 (22.0)	
Rats and cockroaches	35 (9.8)	7 (8.8)	28 (10.1)	
None	198 (55.5)	45 (56.3)	153 (55.2)	
Missing	41 (11.5)	6 (7.5)	35 (12.6)	
**Cigarette smoker at home**				**0.01**
Yes	13 (3.6)	7 (8.8))	6 (2.2)	
No	332 (93.0)	72 (90.0)	260 (93.9)	
Missing	12 (3.4)	1 (1.3)	11 (4.0)	

[a] As determined by chi-squared or Fisher's exact test.
